# Reframing Esophageal Assessment: Integrating FLIP Panometry into Clinical Practice

**DOI:** 10.3390/diagnostics16213565

**Published:** 2026-10-29

**Authors:** Amir Mari, Rita Brun

**Affiliations:** 1 Gastroenterology and Hepatology Unit, Nazareth Hospital EMMS, Nazareth 16100, Israel; 2 Faculty of Medicine, Bar-Ilan University, Ramat Gan 5290002, Israel; 3 Gastroenterology Institute, Rambam Healthcare Campus, Haifa 3109601, Israel; 4 Rappaport Faculty of Medicine, Technion Institute, Haifa 31096, Israel

## 1. Introduction

The functional lumen imaging probe (FLIP) has evolved from a research instrument into an increasingly standardized adjunct for esophageal physiologic assessment. This approach uses impedance planimetry to estimate the cross-sectional area at multiple axial locations while simultaneously measuring intrabag pressure during controlled volumetric distension. From these measurements, FLIP panometry characterizes EGJ opening mechanics, luminal geometry, distensibility, and the contractile response to distension. These outputs are mechanistically distinct from HRM, which evaluates pressure topography during swallowing, and from endoscopy, which primarily defines the mucosal and structural pathology. The Dallas Consensus provides a standardized framework for acquisition, terminology, quality assessment, and interpretation in patients with suspected esophageal motility disorders [1]. Contemporary reviews and clinical practice updates therefore position FLIP panometry as a complementary physiologic test rather than a replacement for HRM [2,3,4].

## 2. Positioning FLIP Panometry in the Diagnostic Pathway

In the diagnostic pathway, FLIP panometry is best used after high-quality endoscopy has excluded a structural or mucosal cause of dysphagia. Its incremental value is greatest when HRM is unavailable, technically unsuccessful, poorly tolerated, or inconclusive or when a manometric diagnosis requires independent evidence of impaired EGJ opening [1,3,4]. Because the FLIP evaluates the response to sustained distension rather than swallow-induced peristalsis, an agreement with HRM is not expected in every patient. Discordance should prompt the integration of symptoms, the endoscopic anatomy, timed barium esophagram findings, and the technical quality of both physiologic studies rather than an automatic prioritization of either test. The European FLIP consensus similarly emphasizes its complementary role in assessing EGJ opening, esophageal wall mechanics, and distension-induced motor activity [4].

The Dallas Consensus recommends a standardized protocol using a 16 cm catheter and stepwise balloon filling at 50, 60, and 70 mL, with each volume maintained for at least 30 s [1]. Interpretation requires a confirmation of the appropriate catheter position and a review of the dynamic study rather than the reliance on a single numerical observation. Principal EGJ metrics include the distensibility index (EGJ-DI), calculated from the cross-sectional area relative to intrabag pressure, and maximal EGJ diameter. Normal EGJ opening is defined by an EGJ-DI ≥ 2.0 mm^2^/mmHg together with a maximal diameter ≥ 16 mm, whereas reduced EGJ opening requires an EGJ-DI < 2.0 mm^2^/mmHg and a maximal diameter < 12 mm [1]. Results between these categories are indeterminate and should not be dichotomized as normal or pathologic. These thresholds are protocol-specific and must be interpreted in the context of the fill volume, pressure stability, catheter position, and overall motility pattern.

Broader adoption will also depend on managing the operator learning curve. Although the Dallas Consensus standardizes acquisition and interpretation, reliable performance still requires proficiency in catheter positioning, recognition of artifacts, selection of stable measurement periods, and integrated assessment of EGJ opening and contractile patterns. Early implementation should therefore include structured training, supervised case review, and local competency assessment, particularly for indeterminate or discordant studies [1].

Contractile activity provides a second interpretive dimension. Repetitive antegrade contractions indicate an organized response to distension and are commonly associated with preserved esophageal motor function. By contrast, absent contractility, diminished contractile responses, repetitive retrograde contractions, or sustained occluding contractions may support a major motor disorder when they occur in conjunction with abnormal EGJ opening and an appropriate clinical phenotype [1,4]. No contractile pattern should be interpreted independently of EGJ mechanics or technical adequacy. FLIP topography studies have demonstrated incremental phenotypic information alongside HRM in achalasia spectrum disorders and other clinically relevant motor presentations [5,6].

### 2.1. Achalasia and Achalasia-like Disorders

FLIP panometry is particularly useful when achalasia or an achalasia-like disorder is suspected despite inconclusive HRM. The combination of reduced EGJ opening and absent or spastic contractility provides physiologic evidence of an obstructive motor phenotype, but it is not etiologically specific and should be interpreted with symptoms, endoscopic findings, HRM, and timed barium esophagram [1,7]. The Chicago Classification v4.0 recommends supportive testing with FLIP or timed barium esophagram when achalasia is suspected despite borderline or normal integrated relaxation pressure. It also requires corroborative evidence of obstruction before manometric EGJOO is considered clinically conclusive [7]. This requirement is important because EGJOO is heterogeneous and may reflect early or variant achalasia, mechanical obstruction, opioid-related dysfunction, hiatal or catheter-related artifacts, or clinically insignificant manometric variation. In a validation cohort of 687 patients, 86% of those with reduced EGJ opening on FLIP assessment had a conclusive EGJ outflow disorder according to the Chicago Classification v4.0, whereas 99% of those with normal EGJ opening had normal EGJ outflow [8].

### 2.2. The Clinical Value of Normal FLIP Panometry

The principal clinical strength of a technically adequate normal study is its exclusionary value for EGJ outflow pathology. Normal EGJ opening together with an appropriate contractile response substantially reduces the probability of achalasia or clinically relevant EGJOO. In an early cohort of 111 patients with normal FLIP panometry, discordant HRM findings were frequently equivocal, and clinically none of the patients were considered to have a major motor disorder [9]. In a study of 722 subjects, 95% of patients classified as having normal FLIP motility had normal motility or ineffective esophageal motility on HRM [10]. A subsequent two-center validation study involving 805 patients reported a 99% negative predictive value for EGJ outflow disorders when FLIP panometry was normal [11]. These estimates are clinically compelling, although predictive values remain dependent on disease prevalence, the referral setting, protocol adherence, and the reference standard used.

Consistent with these findings, the Dallas Consensus states that a major motor disorder is unlikely when FLIP panometry is normal [1]. In carefully selected patients, a technically adequate normal study may therefore reduce the immediate need for HRM. This should be framed as a probability-based decision rather than categorical exclusion: normal FLIP panometry does not rule out every manometric abnormality, explain persistent symptoms, or establish an alternative diagnosis. Ongoing dysphagia, discordant radiographic findings, or high clinical suspicion should prompt complementary evaluations [1,10,11].

## 3. FLIP Panometry During Therapy: Measurement or Guidance?

During laparoscopic Heller myotomy or POEM, FLIP panometry can quantify the immediate change in EGJ opening produced by the disruption of the lower esophageal sphincter. Serial measurements obtained under comparable technical conditions provide an objective physiologic correlate of the intervention. Early studies demonstrated measurable increases in EGJ distensibility during myotomy and suggested that insufficient or excessive post-treatment opening may be clinically relevant [12].

Subsequent observational studies have associated intraoperative FLIP assessment and post-myotomy EGJ distensibility with symptomatic outcomes after POEM or surgical myotomy [13,14]. These associations support biologic plausibility and potential utility for procedural quality assessment. However, reported target ranges vary across protocols, devices, anesthetic conditions, and outcome definitions, and an observed association does not establish a universally applicable therapeutic threshold.

Contemporary clinical practice recommendations therefore state that an intraprocedural FLIP may be considered, when available, during POEM or laparoscopic Heller myotomy to assess the physiologic effect of lower esophageal sphincter disruption [3]. Its role is adjunctive: it may identify an unexpectedly limited change in EGJ opening or provide reassurance that a measurable physiologic effect has occurred.

FLIP-derived measurements should not serve as the sole determinant of the myotomy length or procedural modification. Interpretation requires a stable catheter position; comparable balloon volumes; an awareness of pressure artifacts; and integration with the procedural anatomy, endoscopic findings, symptom phenotype, and operator assessment. Prospective trials are needed to determine whether protocolized FLIP-guided intervention improves outcomes relative to standard procedural judgment.

## 4. Beyond Achalasia

The applicability of FLIP panometry beyond achalasia depends on the pathophysiologic question being addressed. Potential indications include phenotyping EGJOO, evaluating unexplained dysphagia, quantifying esophageal remodeling in eosinophilic esophagitis, characterizing benign strictures, and assessing EGJ mechanics after foregut surgery [2,4,15]. Across these settings, the FLIP should be regarded as a measurement platform rather than a disease-specific diagnostic test; the clinical meaning of a metric depends on the population, protocol, comparator, and outcome of interest.

In eosinophilic esophagitis, the FLIP can quantify the pressure–geometry relationship of the esophageal body and may detect reduced distensibility associated with fibrostenotic remodeling. This biomechanical information is not fully captured by the mucosal appearance or eosinophil density. Nevertheless, the FLIP does not establish diagnoses of eosinophilic esophagitis and should not replace endoscopy with histologic sampling. Its potential roles include characterizing the fibrostenotic burden, assessing persistent obstructive symptoms, and providing a physiologic outcome measure, but clinically actionable thresholds and longitudinal responsiveness require further validation [3,16].

For gastroesophageal reflux disease, the evidentiary basis is less mature. EGJ distensibility may be influenced by the anatomy, sedation, balloon position, prior surgery, and measurement protocol, and a substantial overlap exists between clinical phenotypes. Current recommendations therefore do not support the FLIP as a stand-alone diagnostic test for gastroesophageal reflux disease or as an isolated basis for antireflux intervention [3].

## 5. The Future: Integration Rather than Replacement

Routine implementation should be based on a clearly specified clinical question and should integrate FLIP panometry with established modalities. Endoscopy identifies structural and mucosal disease; HRM defines swallow-associated pressure topography and motor diagnoses; timed barium esophagram assesses bolus retention and emptying; and FLIP panometry quantifies geometry, distensibility, and distension-induced contractility [1,2,3,4]. The tests are therefore complementary rather than interchangeable, and their relative contribution depends on the pretest probability and the source of diagnostic uncertainty.

Technical standardization remains fundamental to internal validity and reproducibility. Clinically important variables include the catheter position across the EGJ, balloon fill volume, duration of observation, patient position, sedation depth and agent, respiratory and vascular artifacts, intrabag pressure stability, and operator experience. Interpretation should document protocol adequacy and should be based on the integrated pattern across filling steps rather than an isolated EGJ-DI value. Because pattern classification incorporates dynamic and partly qualitative judgments, inter-observer variability may be greatest during the learning phase and in borderline or indeterminate studies. Broader dissemination will therefore require structured training, supervised interpretation, periodic calibration against expert-reviewed cases, and local monitoring of agreement and technical adequacy. The Dallas Consensus provides a common framework for these efforts, but competency-based implementation and ongoing quality assurance will remain necessary to limit operator-dependent variation [1,4].

Future studies should prioritize prospective, multicenter designs with prespecified protocols, blinded interpretation, clinically meaningful reference standards, and longitudinal outcomes. Key objectives include external validation of thresholds; reproducibility across devices, positions, and sedation strategies; classification and natural history of indeterminate patterns; the incremental diagnostic value beyond HRM and timed barium esophagram; treatment–effect modification; and cost-effectiveness. Automated analysis and machine learning methods may improve pattern recognition by consistently extracting the EGJ diameter, distensibility, pressure stability, and the timing and direction of the contractile activity across the full filling sequence. Rather than forcing borderline studies into binary categories, future systems could quantify uncertainty, identify the features responsible for an indeterminate classification, and flag technically inadequate or discordant examinations for expert review. Early multicenter models have shown promising performance in classifying EGJ opening and contractile response patterns according to the Dallas Consensus, suggesting the potential to improve consistency and accessibility [15]. However, these tools should support rather than replace clinical interpretation and will require transparent feature definitions, representative training data, external validation across devices and protocols, calibration assessment, and evidence that they improve patient-level decisions before routine adoption.

In conclusion, FLIP panometry provides a distinct physiologic assessment of EGJ opening and the esophageal response to distension. The strongest current evidence supports its use as an adjunct when conventional testing leaves uncertainty about EGJ outflow and as a potentially informative intraprocedural measure during achalasia therapy [1,2,3,4]. A technically adequate normal study substantially lowers the probability of a clinically relevant EGJ outflow disorder, whereas an abnormal study refines—but does not independently establish—the diagnosis or treatment indication. Scientific and clinical progress will depend on protocol standardization; explicit reporting of technical adequacy; prospective outcome validation; and disciplined integration with symptoms, endoscopy, HRM, and radiographic testing.

## Data Availability

No new data were created or analyzed in this editorial. Data sharing is not applicable to this article.
